# Supplementary material for: Lower vaccine-acquired immunity in the elderly population following two-dose BNT162b2 vaccination is alleviated by a third vaccine dose
Source: Nat Commun. 2022 Aug 8;13:4615. doi: 10.1038/s41467-022-32312-1 (PMC9358634; doi:10.1038/s41467-022-32312-1)
Supplement: Supplementary file 1 — Supplementary Information [file 41467_2022_32312_MOESM1_ESM.pdf]

Lower vaccine-acquired immunity in the elderly population following two-dose BNT162b2 vaccination is alleviated by a third vaccine dose

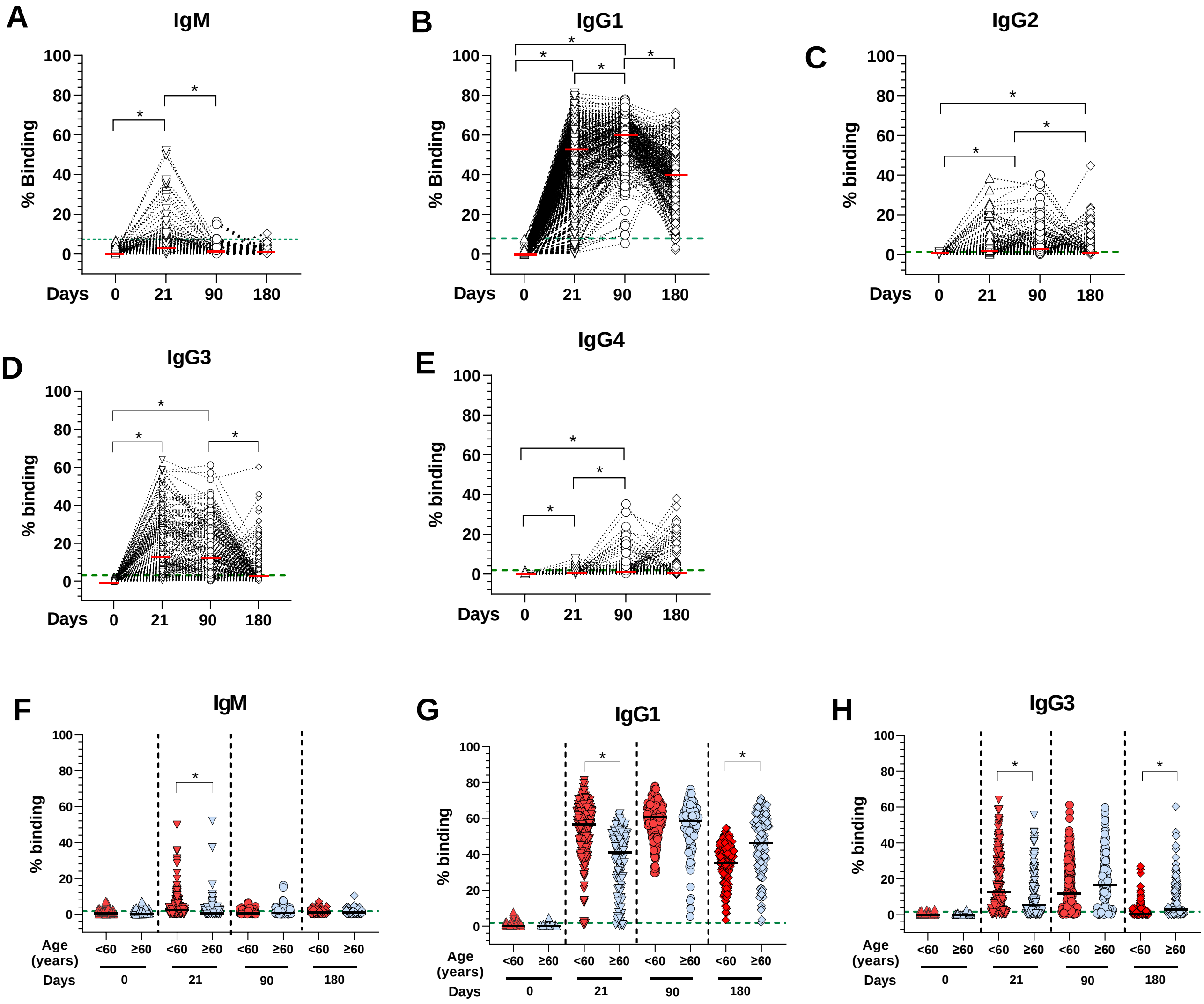

Supplementary Figure 1. Anti-SARS-CoV-2 spike protein antibody isotype response after vaccination. A flow cytometry-based assay using the full Spike protein (SFB) assay was used to determine antibody isotype in the same 312 vaccinated individuals described in Figure 1. (A) IgM. Median of group values at day 0, 21, 90 and 180: was 0.4, 1.04, 0.59 and 0.64%, respectively. Antibody levels below the maximum range (7.385) were considered baseline values . *, p < 0.001, Friedman test; (B) IgG1. Median of group values at day 0, 21, 90 and 180: was 0.12, 51.8, 60 and 39.7%, respectively. Antibody levels below the maximum range (7.893) were considered baseline values. *, p < 0.001, Friedman test; (C) IgG2. Median of values at day 0, 21, 90 and 180: was 0.37, 0.52, 1.3 and 0.3, respectively. Antibody levels below the maximum range (1.44) were considered baseline values. *, p < 0.001, Friedman test; (D) IgG3. Median of values at day 0, 21, 90 and 180: was 0.08, 12.53, 11.8 and 1, respectively. Antibody levels below the maximum range (3.19) were considered baseline values. *, p < 0.001, Friedman test; (E) IgG4. Median of values at day 0, 90 and 180: was 0.03, 0.06, 0.38 and 0.35, respectively. Antibody levels below the maximum range (1.953) were considered baseline values. *, p < 0.001, Friedman test. Median of group values are indicated by a red bar. (F) IgM, (G) IgG1 and (H) IgG3 antibody levels comparison between the analyzed age groups: <60 (n=186) and ≥60 (n=144), at different time points (day 0, 21 and 90). The Median of group values are represented as a black line through the dots. The red lines or boxes represent the maximum range of the samples. *, p < 0.001, Mann Whitney test.

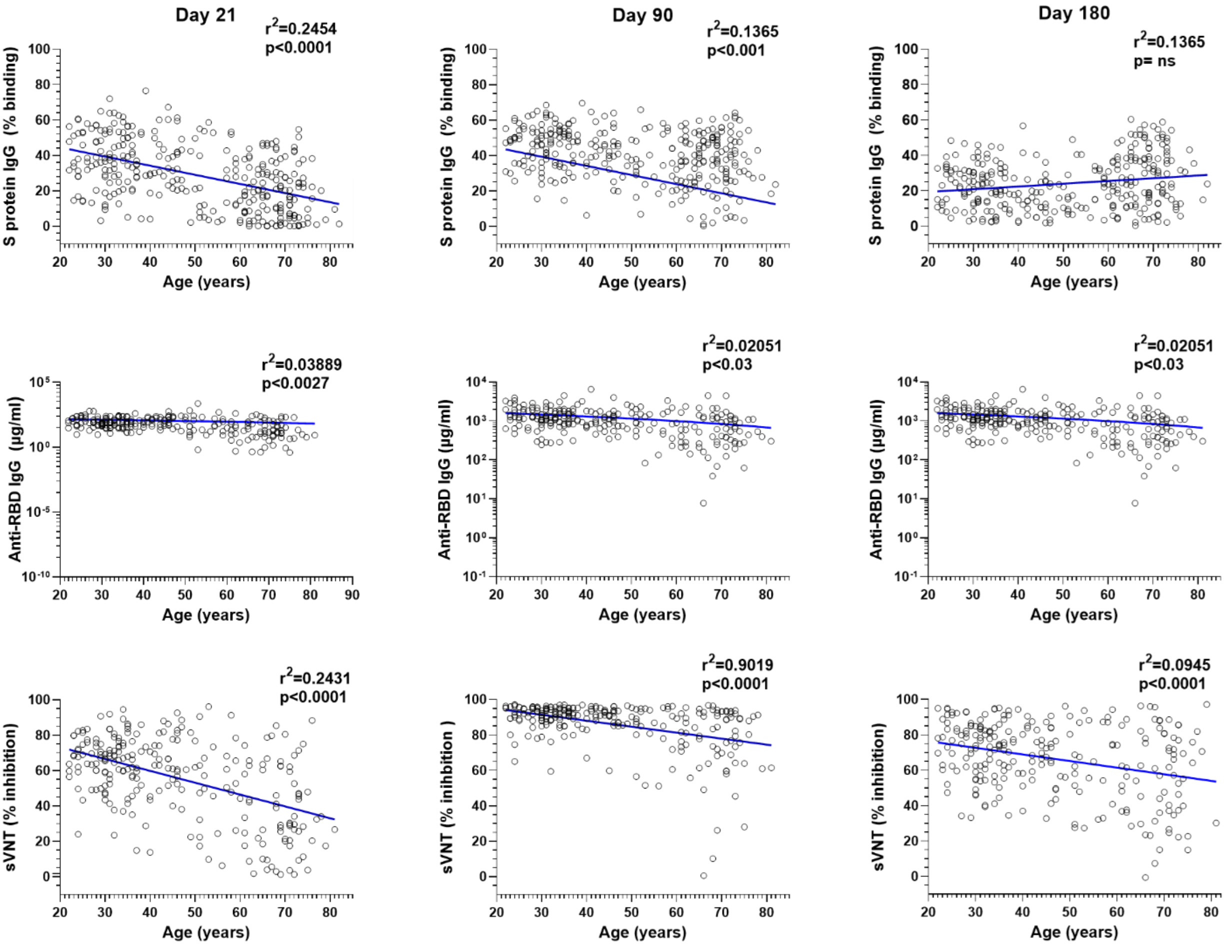

Supplementary Figure 2. Linear correlations between participant's age and SARS-CoV-2 specific antibody titer measured at different time after vaccination. Correlation was analyzed by simple linear regression at day 21, 90 and 180 for the Flow cytometry-based assay for the S-pike protein (SFB) (Higher panels); the commercial RBD Roche S assay (mid panels) ; and the sVNT assay (Lower panels).

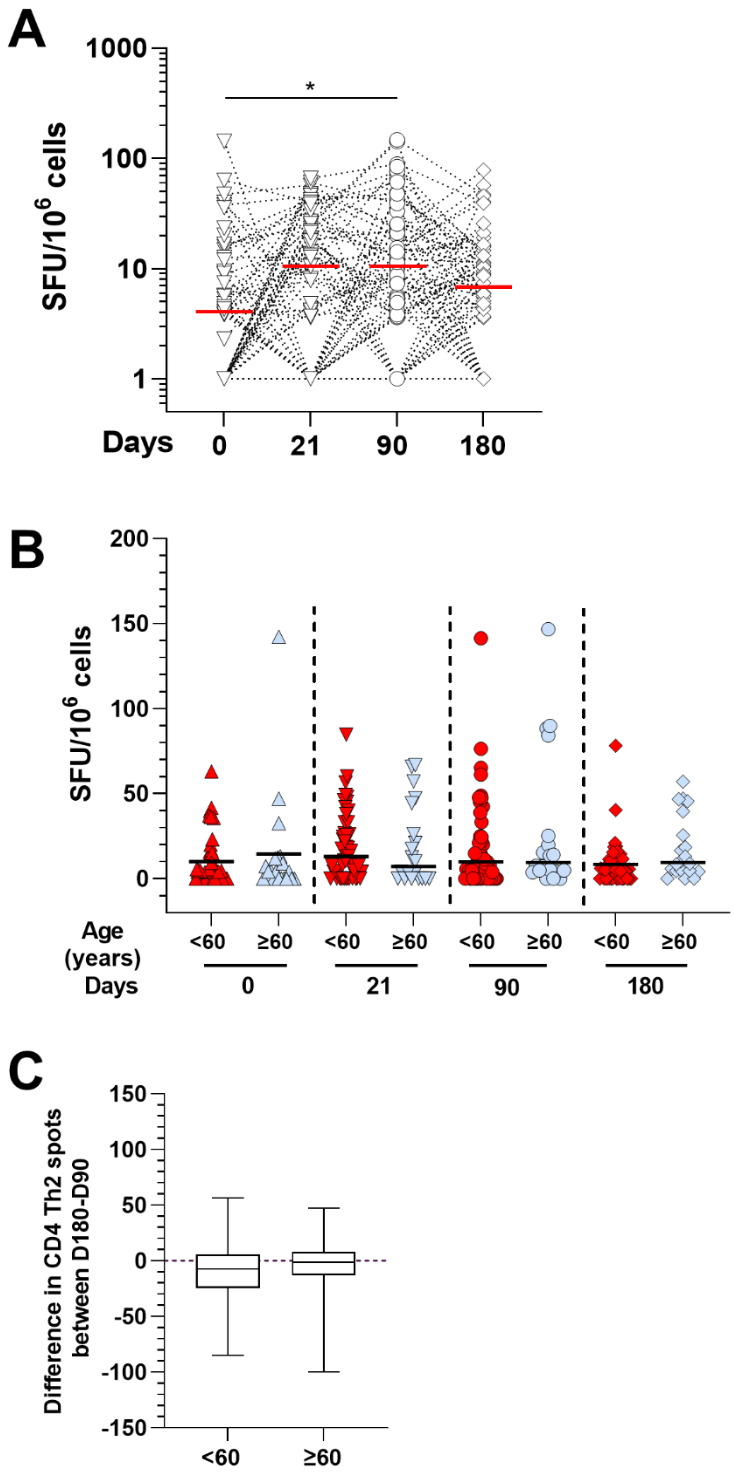

Supplementary Figure 3. CD4 Th2 responses. (A) Kinetics of Spike-protein-specific Th2 cells overtime in vaccinees. CD4 Th2 cells were assayed on a subset of vaccinees \on a subset of vaccinees (n =71) by IL4/IL5/IL13 ELISPOT using 15 mer pool peptides. Data are presented are spot forming units (SFU) per million of PBMC from paired samples from vaccinated individuals at 4 time points. Each data point represents the normalized mean spot count from duplicate wells for one study participant, after subtraction of the medium-only control . The median values of each group are represented by a red line and were at day 0, 21, 90 and 180: 4.1, 10.4, 10 and 7.4 SFU for CD4 Th2 T cells. *, p < 0.01, (B) Comparison between the analyzed age groups at day 0, 21 , 90 and 180, (n=83), <60: (n= 51) and ≥60 (n=20). Median values are indicated by a dark line. *, p < 0.01Mann Whitney test. *, p < 0.01, Mann Whitney test. (C) Box plots (with whiskers) showing difference in CD4 Th2 cell responses measure in the different assays between days 180 and 90 for paired samples for both age groups, <60: (n= 51), and ≥60 (n=20). p = 0.003, Mann Whitney test.

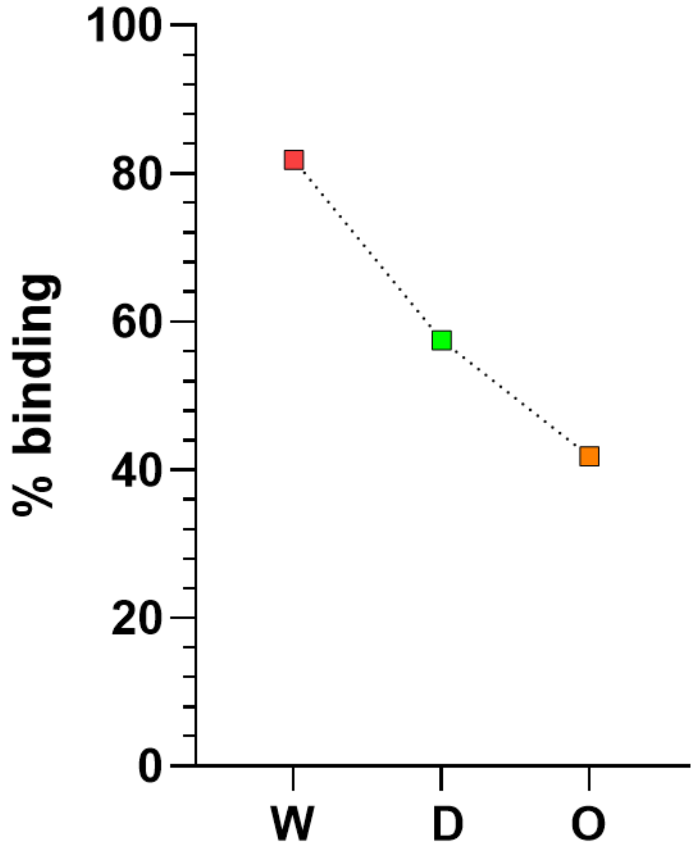

Supplementary Figure 4. Detection of expression the Spike protein from different strains of SARS-CoV-2 on the surface of HEK203 transduced cells. The flow cytometry-based assay using the full Spike protein (SFB) assay was used as described in Figure 1. The serum used to detect S protein expression of different SARS-CoV-2 (W, Wuhan; D, Delta; and O, Omicron (BA.1) strains) was from an individual who recovered from a previous infection and then was vaccinated with two doses of the BNT162b2 vaccine and taken at 90 days postsecond doses.

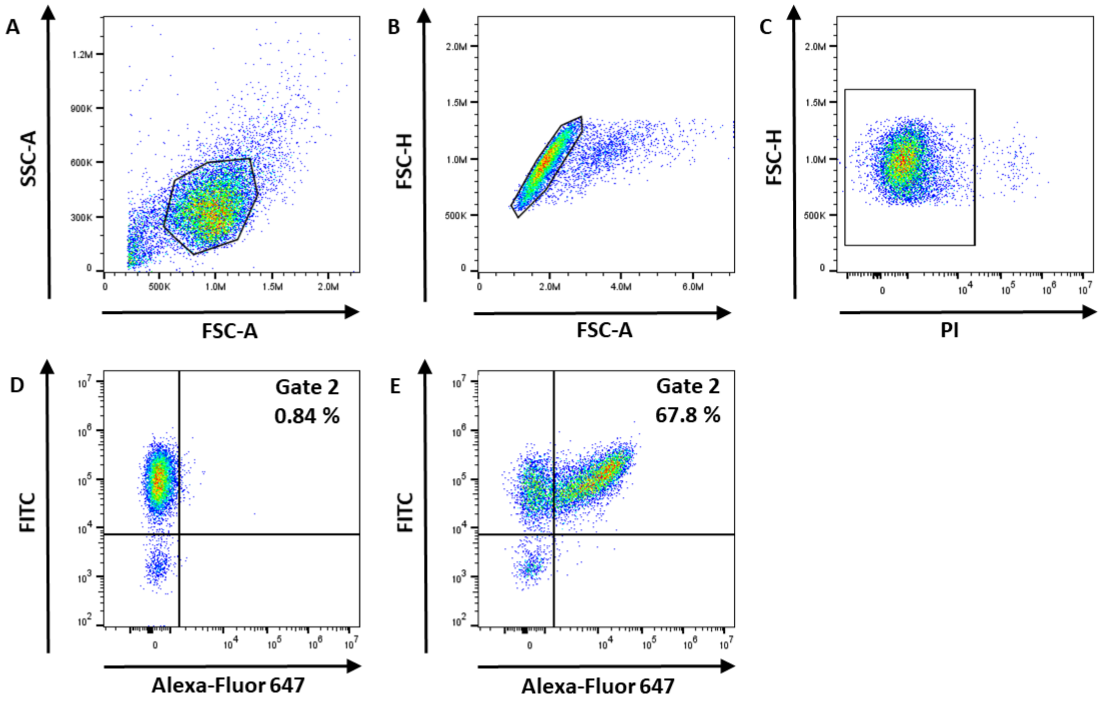

Supplementary Figure 5. Representative gating strategy for SFB assay to determine spike-specific antibody response. Cells were gated on: (A) FSC-A/SSC-A to exclude cell debris, (B) FSC-A/FSC-H to select for single cells, (C) FSC-H/PI to select for live cells (PI-negative population), (D, E) FITC/Alexa Fluor 647. Binding is determined by the percentage of GFP-positive S protein-expressing cells that are bound by specific antibody, indicated by the events that are Alexa Fluor 647- and FITC-positive (Gate 2). (D) Healthy control individual plasma, 1:100 diluted; (E) COVID-19 patient plasma, 1:100 diluted

Supplementary Table 1. Characteristics of the study population

Characteristics	<60 years	≥60 years	Total cohort number (%)
**Total**	178 (57.1)	134 (42.9)	312
**Gender**			
Male (%)	51 (28.3)	79 (61.7)	130 (41.7)
Female (%)	127 (69.7)	55 (30.3)	182 (58.3)
**Ethnicity**			
Chinese (%)	106 (46.9)	120 (53.1)	226 (72.4)
Indians (%)	14 (60.9)	9 (39.1)	23 (7.6)
Malays (%)	26 (89.7)	3 (10.3)	29 (8.8)
Others (%)	32 (94.1)	2 (5.9)	34 (11.2)
Median years (minimum-maximum)	35 (22-59)	69 (60-82)	50.85 (22-82)

Supplementary Table 2. Immune reactivity across timepoints

Assays	Proportion of volunteers with response above individual baseline * (%)		
	At day 21	At day 90	At day 180
Anti-spike protein IgG (SFB)	306/312 (98)	311/312 (99)	311/312 (99)
Anti-RBD IgG (Roche S)	310/312 (99.3)	312/312 (100)	312/312 (100)
sVNT	247/312 (79.1)	309/312 (99)	293/312 (93.9)
B cell Elispot	17/35 (48.6)	28/35 (80)	30/35 (85.7)
IL-2 T cells	153/155 (98.7)	155/155 (100)	154/155 (99.3)
IFNγ T cells	144/155 (92.9)	149/155 (96.1)	153/155 (98.7)
CD8 T cell Elispot	39/72 (54.2)	54/72 (75)	29/72 (40.3)
CD4 Th1 Elispot	54/78 (69.2)	66/78 (84.6)	65/78 (83.3)
CD4 Th2 Elispot	42/71 (59)	42/71 (59)	35/71 (49)

* Paired samples

Supplementary Table 3. Long-term immune responses in vaccinated individuals

Assays	Proportion of individuals with a lower response at day 180 compared with day 90 (%)*			
	Total population	< 60 years	≥60 years	*P value***
Anti-spike protein IgG (SFB)	243/312 (78)	176/178 (98.9)	66/134 (49.2)	0.0001
Anti-RBD IgG (Roche S)	243/312 (77.8)	153/178 (85.9)	89/134 (66.4)	0.0001
sVNT	243/312 (77.5)	119/178 (66.8)	123/134 (91.8)	NS
B cell Elispot	10/34 (29.4)	3/16 (18.75)	7/18 (52.9)	NS
IL-2 T cells	97/155 (62.5)	56/81 (69.1)	40/74 (54)	NS
IFNγ T cells	72/155 (46.5)	49/81 (60.4)	24/74 (32)	0.0003
CD8 T cell Elispot	45/72 (67.9)	31/45 (68.9)	13/27 (48.1)	NS
CD4 Th1 Elispot	37/79 (46.8)	21/51 (41.2)	16/28 (57.1)	NS
CD4 Th2 Elispot	35/71 (50.7)	30/51 (58.2)	11/20 (55)	NS

* Paired samples

** Difference between age groups

Supplementary Table 4. Low responders as determined by the SFB assay

Days	21		90		180	
Median antibody response (% binding)	28.3%		41.98%		23.35%	
Proportion of low responders* (n / tested number)	-		37.2 (116/312)		22.2 (88/312)	
Age (Years)	N	% of total cohort	N	% of total cohort	N	% of total cohort
<60	57	18.2	42	13.5	40	12.8
>60	99	31.7	74	23.7	48	13.5

* Low responders are defined as fully vaccinated individuals with antibody response below cohort's median response at consecutive timepoints (%). For example, the low responders, at day 90, have antibody response below cohort's median response at both day 21, 90 and 180.

Supplementary Table 5. Low responders as determined by Roche S

Days	21		90		180	
Median antibody response (U/ml)	50.28		927		677	
Proportion of low responders* (n/tested number)	-		36.5 (114/312)		31.4 (98/312)	
Age (Years)	N	% of total cohort	N	% of total cohort	N	% of total cohort
<60	60	19.2	32	10.25	26	8.3
>60	99	31.7	82	26.2	69	22

* These values were determined as in Supplementary table 4.

Supplementary Table 6. Low responders as determined by sVNT

Days	21		90		180	
Median inhibition (%I)	56.39		89.87		67.4	
Proportion of low responders* (n/tested number)	-		39.1 (122/312)		34.9 (109/312)	
Age (Years)	N	% of total cohort	N	% of total cohort3	N	% of total cohort
<60	53	16.9	38	12.18	35	11.2
>60	104	33	84	26.9	74	23.7

* These values were determined as in Supplementary table 4.

Supplementary Table 7. Low responders as determined by measurement of IL-2 production by T cells

Days	21		90		180	
Median antibody response (pg/ml)	54.48		89.39		78.06	
Proportion of low responders* (n / tested number)	-		29.7 (46/155)		25.2 (39/155)	
Age (Years)	N	% of total cohort	N	% of total cohort	N	% of total cohort
<60	40	25.8	25	16.1	21	13.5
>60	37	23.9	21	13.5	18	11.6

* These values were determined as in Supplementary table 4.

Supplementary Table 8. Low responders as determined by measurement of IFN-γ production by T cells

Days	21		90		180	
Median antibody response (U/ml)	11.69		33.81		32.43	
Proportion of low responders* (n / tested number)	-		32.2 (50/155)		26.5 (41/155)	
Age (Years)	N	% of total cohort	N	% of total cohort	N	% of total cohort
<60	33	21.2	22	14.2	20	12.9
>60	45	29	28	18	21	13.5

* These values were determined as in Supplementary table 4. The median values were at day 21: 11.69 pg/ml, at day 90: 33.81 pg/ml and day 180: 32.43 pg/ml).

Supplementary Table 9. Low responders as determined by CD4 Th1 ELISPOT

Days	21		90		180	
Median antibody response (Spot forming units)	53.48		89.39		78.06	
Proportion of low responders* (n / tested number)	-		19.2 (15/78)		6.4 (5/78)	
Age (Years)	N	% of total cohort	N	% of total cohort	N	% of total cohort
<60	22	28.2	8	10.2	1	1.2
>60	13	16.7	7	9	4	6.4

* These values were determined as in Supplementary table 4.

Supplementary Table 10. Low responders as determined by CD8 ELISPOT

Days	21		90		180	
Median antibody response (Spot forming units)	44.85		71.65		180.42	
Proportion of low responders* (n / tested number)	-		36 (26/72)		23.6 (17/72)	
Age (Years)	N	% of total cohort	N	% of total cohort	N	% of total cohort
<60	25	42.3	18	11.5	13	1.2
>60	11	57.7	8	7.7	4	6.4

* These values were determined as in Table S4. The median values were at day 21: 44.85 SFU, at day 90: 71.65 SFU and day 180: 42.1 SFU).

Supplementary Table 11: Spike protein peptide pool utilized in whole blood cytokine release assay

Spike peptide pool			
Amino Acid Sequence	Amino Acid Position	Amino Acid Sequence	Amino Acid Position
IRGWIFGTTLDSKTQ	101-115	SIIAYTMSLGAENSV	691-705
FGTTLDSKTQSLLIV	106-120	TMSLGAENSVAYSNN	696-710
CTFEYVSQPFLMDLE	166-180	STECSNLLLQYGSFC	746-760
VSQPFLMDLEGKQGN	171-185	NLLLQYGSFCTQLNR	751-765
TRFQTLLALHRSYLT	236-250	KNTQEVFAQVKQIYK	776-790
LLALHRSYLTPGDSS	241-255	VFAQVKQIYKTPPIK	781-795
RSYLTPGDSSSGWTA	246-260	KQIYKTPPIKDFGGF	786-800
CALDPLSETKCTLKS	291-305	TPPIKDFGGFNFSQI	791-805
LSETKCTLKSFTVEK	296-310	NFSQILPDPSKPSKR	801-815
CTLKSFTVEKGIYQT	301-315	AGFIKQYGDCLGDIA	831-845
FTVEKGIYQTSNFRV	306-320	QYGDCLGDIAARDLI	836-850
GIYQTSNFRVQPTES	311-325	GAALQIPFAMQMAYR	891-905
SASFSTFKCYGVSPT	371-385	QMAYRFNGIGVTQNV	901-915
TFKCYGVSPTKLNDL	376-390	FNGIGVTQNVLYENQ	906-920
YNYKLPDDFTGCVIA	421-435	DSLSSTASALGKLQD	936-950
WNSNNLDSKVGGNYN	436-450	TASALGKLQDVVNQN	941-955
LDSKVGGNYNYLYRL	441-455	AQALNTLVKQLSSNF	956-970
GGNYNYLYRLFRKSN	446-460	VLNDILSRLDKVEAE	976-990
YLYRLFRKSNLKPFE	451-465	LITGRLQSLQTYVTQ	996-1010
FRKSNLKPFERDIST	456-470	QLIRAAEIRASANLA	1011-1025
LKPFERDISTEIYQA	461-475	AEIRASANLAATKMS	1016-1030
GPKKSTNLVKNKCVN	526-540	APHGVVFLHVTYVPA	1056-1070
TNLVKNKCVNFNFNG	531-545	HWFVTQRNFYEPQII	1101-1115
FNFNGLTGTGVLTES	541-555	KEIDRLNEVAKNLNE	1181-1195
LTGTGVLTESNKKFL	546-560	LNEVAKNLNESLIDL	1186-1200
RAGCLIGAEHVNNSY	646-660	KNLNESLIDLQELGK	1191-1205
IGAEHVNNSYECDIP	651-665	IWLGFIAGLIAIVMV	1216-1230
SVASQSIIAYTMSLG	686-700		

Supplementary Table 12. Spike peptides (n=211) for CD8 ELISPOT

LTDEMIAQY	GQTGKIADY	NIDGYFKIY	SVYAWNRKR
VRFPNITNL	YRLFRKSNL	HVTYVPAQEK	GLTVLPPLL
VRFPNITNL	VRDPQTLEI	YYVGYLQPRTF	IYQTSNFRV
RLFRKSNLK	VYSTGSNVF	RFDNPVLPF	KFLPFQQF
VRFPNITNL	WTAGAAAYY	YLQPRTFLL	SLIDLQELGK
VYDPLQPEL	GVYYHKNNK	TRFASVYAW	KWPWYIWLGF
YLQPRTFLL	VRDPQTLEI	LVRDLPQGF	TRFASVYAW
LLTDEMIAQY	HLMSFPQSA	IVRFPNITNL	SGWTAGAAAYY
VLKGVKLHY	YYHKNNKSW	YLQPRTFLL	VFKNIDGYF
VLNDILSRL	RLQSLQTYV	NSFTRGVYY	ILPDPSKPSK
VYSTGSNVF	FRVQPTESI	FRKSNLKPF	QRNFYEPQI
IRGDEVRQI	RFPNITNLCPF	YKTPPIKDF	ALNTLVKQL
TRFQTLLAL	LVKQLSSNF	CVADYSVLY	VFVSNGTHWF
QIYKTPPIK	INITRFQTL	YKTPPIKDF	LPQGFSAL
TRFQTLLAL	YQPYRVVVL	VYYHKNNKSW	QPYRVVVL
RQIAPGQTGK	TDEMIAQY	NLTTRTQL	SIIAYTMSL
TYVPAQEKNF	TQLPPAYTNSF	SIVRFPNITNL	YRVVVLSF
GVYFASTEK	LQIPFAMQMAY	YQPYRVVVL	LLALHRSYL
TLKSFTVEK	NTSNQVAVLY	KRFDNPVL	GPKKSTNL
QYIKWPWYI	PLLTDEMIAQY	QIAPGQTGK	VYYPDKVF
YLQPRTFLL	LITGRLQSL	SKHTPINLV	IYSKHTPINL
TRTQLPPAY	IRGDEVRQI	LTDEMIAQY	SAPHGVVFL
TRFQTLLAL	KPFERDISTEI	SPRRARSV	CYFPLQSYGF
KQIYKTPPIK	LQIPFAMQM	NYNYLYRLF	KVTLADAGFIK
EPVLKGVKL	EYVSQPFLM	KCYGVSPTK	RVYSTGSNVF
TLDSKTQSL	YTNSFTRGVYY	HWFVTQRNF	VYDPLQPEL
VYSSANNCTF	YQPYRVVVL	YSSANNCTFEY	NLNESLIDL
LYNSASFSTF	FRKSNLKPF	FIAGLIAIV	GYLQPRTFL
KRFDNPVLPF	KRFDNPVLPF	VYYPDKVF	GVYYPDKVF
VYDPLQPEL	YTNSFTRGVY	QSAPHGVVF	YYHKNNKSW
IYKTPPIKDF	NQKLIANQF	YSKHTPINL	ALDPLSETK
YYVGYLQPRTF	YRLFRKSNL	VVFLHVTYV	YFPLQSYGF
TRTQLPPAY	QLTPTWRVY	VYDPLQPEL	EPVLKGVKL
STECSNLLLQY	TRTQLPPAY	DLLFNKVTL	FPQSAPHGVVF
VRDPQTLEI	LLFNKVTL	KRFDNPVL	SIVRFPNITNL
TLDSKTQSL	VASQSIIAY	YPDKVFRSSVL	TTEILPVSMTK
FRVQPTESI	FRVQPTESI	RFDNPVLPF	VRDPQTLEIL
VYDPLQPEL	VFVSNGTHW	YKTPPIKDF	IGAEHVNNSY
TSNQVAVLY	NLDSKVGGNY	PYRVVVLSF	FRSSVLHST
KIADYNYKL	TEILPVSMTK	YRLFRKSNL	GINASVVNIQK
GRLQSLQTY	KCTLKSFTVEK	KTSVDCTMY	KSTNLVKNK
GRLQSLQTY	YLQPRTFLLK	ASANLAATK	KMSECVLGQSK
VTYVPAQEK	NITRFQTL	GRLQSLQTY	FQPTNGVGY
NYNYLYRLF	TLLALHRSY	YPDKVFRSSV	QYIKWPWYIW
SPRRARSVA	IVRFPNITNL	VADYSVLY	GVVFLHVTY
QPYRVVVL	ARSVASQSI	RLNEVAKNL	GPKKSTNLV
KVFRSSVLH	LLFNKVTLA	SLGAENSVAY	RVDFCGKGY
YFPLQSYGF	GAEHVNNSY	KLNDLCFTNVY	TPINLVRDL
LIDLQELGKY	SSANNCTFEY	YLQPRTFLL	AHFPREGVF
RLDKVEAEV	QYGSFCTQL	YTNSFTRGV	TLKSFTVEK
LADAGFIKQY	KRFDNPVLPF	GPKKSTNL	ASVYAWNRK
RVYSTGSNVF	FRKSNLKPF	SVASQSIIAY	FQFCNDPFL
YLQPRTFL	SEPVLKGVKL	NSFTRGVYY	

Peptide pool adapted from Zhang et al,(2021) and from Kared .(2021).

Supplementary Table 13. Spike peptides (n=315) for CD4 ELISPOT

MFVFLVLLPLVSSQC	DSSSGWTAGAAAYYV	YQPYRVVVLSFELLH	GSFCTQLNRALTGIA
LVLLPLVSSQCVNLT	GWTAGAAAYYVGYLQ	RVVVLSFELLHAPAT	TQLNRALTGIAVEQD
PLVSSQCVNLTTRTQ	GAAAYYVGYLQPRTF	LSFELLHAPATVCGP	RALTGIAVEQDKNTQ
SQCVNLTTRTQLPPA	YYVGYLQPRTFLLKY	LLHAPATVCGPKKST	GIAVEQDKNTQEVFA
NLTTRTQLPPAYTNS	YLQPRTFLLKYNENG	PATVCGPKKSTNLVK	EQDKNTQEVFAQVKQ
RTQLPPAYTNSFTRG	RTFLLKYNENGTITD	CGPKKSTNLVKNKCV	NTQEVFAQVKQIYKT
PPAYTNSFTRGVYYP	LKYNENGTITDAVDC	KSTNLVKNKCVNFNF	VFAQVKQIYKTPPIK
TNSFTRGVYYPDKVF	ENGTITDAVDCALDP	LVKNKCVNFNFNGLT	VKQIYKTPPIKDFGG
TRGVYYPDKVFRSSV	ITDAVDCALDPLSET	KCVNFNFNGLTGTGV	YKTPPIKDFGGFNFS
YYPDKVFRSSVLHST	VDCALDPLSETKCTL	FNFNGLTGTGVLTES	PIKDFGGFNFSQILP
KVFRSSVLHSTQDLF	LDPLSETKCTLKSFT	GLTGTGVLTESNKKF	FGGFNFSQILPDPSK
SSVLHSTQDLFLPFF	SETKCTLKSFTVEKG	TGVLTESNKKFLPFQ	NFSQILPDPSKPSKR
HSTQDLFLPFFSNVT	CTLKSFTVEKGIYQT	TESNKKFLPFQQFGR	ILPDPSKPSKRSFIE
DLFLPFFSNVTWFHA	SFTVEKGIYQTSNFR	KKFLPFQQFGRDIAD	PSKPSKRSFIEDLLF
PFFSNVTWFHAIHVS	EKGIYQTSNFRVQPT	PFQQFGRDIADTTDA	SKRSFIEDLLFNKVT
NVTWFHAIHVSGTNG	YQTSNFRVQPTESIV	FGRDIADTTDAVRDP	FIEDLLFNKVTLADA
FHAIHVSGTNGTKRF	NFRVQPTESIVRFPN	IADTTDAVRDPQTLE	LLFNKVTLADAGFIK
HVSGTNGTKRFDNPV	QPTESIVRFPNITNL	TDAVRDPQTLEILDI	KVTLADAGFIKQYGD
TNGTKRFDNPVLPFN	SIVRFPNITNLCPFG	RDPQTLEILDITPCS	ADAGFIKQYGDCLGD
KRFDNPVLPFNDGVY	FPNITNLCPFGEVFN	TLEILDITPCSFGGV	FIKQYGDCLGDIAAR
NPVLPFNDGVYFAST	TNLCPFGEVFNATRF	LDITPCSFGGVSVIT	YGDCLGDIAARDLIC
PFNDGVYFASTEKSN	PFGEVFNATRFASVY	PCSFGGVSVITPGTN	LGDIAARDLICAQKF
GVYFASTEKSNIIRG	VFNATRFASVYAWNR	GGVSVITPGTNTSNQ	AARDLICAQKFNGLT
ASTEKSNIIRGWIFG	TRFASVYAWNRKRIS	VITPGTNTSNQVAVL	LICAQKFNGLTVLPP
KSNIIRGWIFGTTLD	SVYAWNRKRISNCVA	GTNTSNQVAVLYQDV	QKFNGLTVLPPLLTD
IRGWIFGTTLDSKTQ	WNRKRISNCVADYSV	SNQVAVLYQDVNCTE	GLTVLPPLLTDEMIA
IFGTTLDSKTQSLLI	RISNCVADYSVLYNS	AVLYQDVNCTEVPVA	LPPLLTDEMIAQYTS
TLDSKTQSLLIVNNA	CVADYSVLYNSASFS	QDVNCTEVPVAIHAD	LTDEMIAQYTSALLA
KTQSLLIVNNATNVV	YSVLYNSASFSTFKC	CTEVPVAIHADQLTP	MIAQYTSALLAGTIT
LLIVNNATNVVIKVC	YNSASFSTFKCYGVS	PVAIHADQLTPTWRV	YTSALLAGTITSGWT

LLALHRSYLTPGDSS	QSYGFQPTNGVGYQP	DSTECSNLLLQYGSF	ITGRLQSLQTYVTQQ
HRSYLTPGDSSSGWT	FQPTNGVGYQPYRVV	CSNLLLQYGSFCTQL	LQSLQTYVTQQLIRA
LTPGDSSSGWTAGAA	NGVGYQPYRVVVLSF	LLQYGSFCTQLNRAL	QTYVTQQLIRAAEIR
TQQLIRAAEIRASAN	KNFTTAPAICHDGKA	VYDPLQPELDSFKEE	QELGKYEQYIKWPWY
IRAAEIRASANLAAT	TAPAICHDGKAHFPR	LQPELDSFKEELDKY	KYEQYIKWPWYIWLG
EIRASANLAATKMSE	ICHDGKAHFPREGVF	LDSFKEELDKYFKNH	YIKWPWYIWLGFIAG
SANLAATKMSECVLG	GKAHFPREGVFVSNG	KEELDKYFKNHTSPD	PWYIWLGFIAGLIAI
AATKMSECVLGQSKR	FPREGVFVSNGTHWF	DKYFKNHTSPDVDLG	WLGFIAGLIAIVMVT
MSECVLGQSKRVDFC	GVFVSNGTHWFVTQR	KNHTSPDVDLGDISG	IAGLIAIVMVTIMLC
VLGQSKRVDFCGKGY	SNGTHWFVTQRNFYE	SPDVDLGDISGINAS	IAIVMVTIMLCCMTS
SKRVDFCGKGYHLMS	HWFVTQRNFYEPQII	DLGDISGINASVVNI	MVTIMLCCMTSCCSC
DFCGKGYHLMSFPQS	TQRNFYEPQIITTDN	ISGINASVVNIQKEI	MLCCMTSCCSCLKGC
KGYHLMSFPQSAPHG	FYEPQIITTDNTFVS	NASVVNIQKEIDRLN	MTSCCSCLKGCCSCG
LMSFPQSAPHGVVFL	QIITTDNTFVSGNCD	VNIQKEIDRLNEVAK	CSCLKGCCSCGSCCK
PQSAPHGVVFLHVTY	TDNTFVSGNCDVVIG	KEIDRLNEVAKNLNE	KGCCSCGSCCKFDED
PHGVVFLHVTYVPAQ	FVSGNCDVVIGIVNN	RLNEVAKNLNESLID	SCGSCCKFDEDDSEP
VFLHVTYVPAQEKNF	NCDVVIGIVNNTVYD	VAKNLNESLIDLQEL	CCKFDEDDSEPVLKG
VTYVPAQEKNFTTAP	VIGIVNNTVYDPLQP	LNESLIDLQELGKYE	DEDDSEPVLKGVKLHYT
PAQEKNFTTAPAICH	VNNTVYDPLQPELDS	LIDLQELGKYEQYIK	

NNATNVVIKVCEFQF	SFSTFKCYGVSPTKL	HADQLTPTWRVYSTG	LLAGTITSGWTFGAG
NVVIKVCEFQFCNDP	FKCYGVSPTKLNDLC	LTPTWRVYSTGSNVF	TITSGWTFGAGAALQ
KVCEFQFCNDPFLGV	GVSPTKLNDLCFTNV	WRVYSTGSNVFQTRA	GWTFGAGAALQIPFA
FQFCNDPFLGVYYHK	TKLNDLCFTNVYADS	STGSNVFQTRAGCLI	GAGAALQIPFAMQMA
NDPFLGVYYHKNNKS	DLCFTNVYADSFVIR	NVFQTRAGCLIGAEH	ALQIPFAMQMAYRFN
LGVYYHKNNKSWMES	TNVYADSFVIRGDEV	TRAGCLIGAEHVNNS	PFAMQMAYRFNGIGV
YHKNNKSWMESEFRV	ADSFVIRGDEVRQIA	CLIGAEHVNNSYECD	QMAYRFNGIGVTQNV
NKSWMESEFRVYSSA	VIRGDEVRQIAPGQT	AEHVNNSYECDIPIG	RFNGIGVTQNVLYEN
MESEFRVYSSANNCT	DEVRQIAPGQTGKIA	NNSYECDIPIGAGIC	IGVTQNVLYENQKLI
FRVYSSANNCTFEYV	QIAPGQTGKIADYNY	ECDIPIGAGICASYQ	QNVLYENQKLIANQF
SSANNCTFEYVSQPF	GQTGKIADYNYKLPD	PIGAGICASYQTQTN	YENQKLIANQFNSAI
NCTFEYVSQPFLMDL	KIADYNYKLPDDFTG	GICASYQTQTNSPRR	KLIANQFNSAIGKIQ
EYVSQPFLMDLEGKQ	YNYKLPDDFTGCVIA	SYQTQTNSPRRARSV	NQFNSAIGKIQDSLS
QPFLMDLEGKQGNFK	LPDDFTGCVIAWNSN	QTNSPRRARSVASQS	SAIGKIQDSLSSTAS
MDLEGKQGNFKNLRE	FTGCVIAWNSNNLDS	PRRARSVASQSIIAY	KIQDSLSSTASALGK
GKQGNFKNLREFVFK	VIAWNSNNLDSKVGG	RSVASQSIIAYTMSL	SLSSTASALGKLQDV
NFKNLREFVFKNIDG	NSNNLDSKVGGNYNY	SQSIIAYTMSLGAEN	TASALGKLQDVVNQN
LREFVFKNIDGYFKI	LDSKVGGNYNYLYRL	IAYTMSLGAENSVAY	LGKLQDVVNQNAQAL
VFKNIDGYFKIYSKH	VGGNYNYLYRLFRKS	MSLGAENSVAYSNNS	QDVVNQNAQALNTLV
IDGYFKIYSKHTPIN	YNYLYRLFRKSNLKP	AENSVAYSNNSIAIP	NQNAQALNTLVKQLS
FKIYSKHTPINLVRD	YRLFRKSNLKPFERD	VAYSNNSIAIPTNFT	QALNTLVKQLSSNFG
SKHTPINLVRDLPQG	RKSNLKPFERDISTE	NNSIAIPTNFTISVT	TLVKQLSSNFGAISS
PINLVRDLPQGFSAL	LKPFERDISTEIYQA	AIPTNFTISVTTEIL	QLSSNFGAISSVLND
VRDLPQGFSALEPLV	ERDISTEIYQAGSTP	NFTISVTTEILPVSM	NFGAISSVLNDILSR
PQGFSALEPLVDLPI	STEIYQAGSTPCNGV	SVTTEILPVSMTKTS	ISSVLNDILSRLDKV
SALEPLVDLPIGINI	YQAGSTPCNGVEGFN	EILPVSMTKTSVDCT	LNDILSRLDKVEAEV
PLVDLPIGINITRFQ	STPCNGVEGFNCYFP	VSMTKTSVDCTMYIC	LSRLDKVEAEVQIDR
LPIGINITRFQTLLA	NGVEGFNCYFPLQSY	KTSVDCTMYICGDST	DKVEAEVQIDRLITG
INITRFQTLLALHRS	GFNCYFPLQSYGFQP	DCTMYICGDSTECSN	AEVQIDRLITGRLQS
RFQTLLALHRSYLTP	YFPLQSYGFQPTNGV	YICGDSTECSNLLLQ	IDRLITGRLQSLQTY

Peptide pool adapted from Cassaniti et al (2021).

Supplementary References

1. Zhang et al. Profiling CD8 + T cell epitopes of COVID-19 convalescents reveals reduced cellular immune responses to SARS-CoV-2 variants. *Cell Rep*. **36**, 109708 (2021).

2. Kared et al, SARS-CoV-2-specific CD8+ T cell responses in convalescent COVID-19 individuals. *J. Clin. Invest*. **131**, e145476 (2021).

3. Cassaniti *et al.* SARS-CoV-2 specific T-cell immunity in COVID-19 convalescent patients and unexposed controls measured by ex vivo ELISpot assay. *Clin. Microbiol. Infect.* **27**,1029-1034 (2021).

§ **SCOPE cohort study group**

Anthony Torres Ruesta[1], Vanessa Neo[1], Wendy Yehui Chen[1], Estelle Yi Wei Goh[1], Alice Soh Meoy Ong[1], Adeline Chiew Yen Chua[1], Samantha Yee Teng Nguee[1], Yong Jie Tan[1], Weiyi Tang[1].

[1]A*STAR Infectious Diseases Labs (A*STAR ID Labs), Agency for Science, Technology and Research (A*STAR), Singapore
