# Supplementary material for: Lower vaccine-acquired immunity in the elderly population following two-dose BNT162b2 vaccination is alleviated by a third vaccine dose
Source: Nat Commun. 2022 Aug 8;13:4615. doi: 10.1038/s41467-022-32312-1 (PMC9358634; doi:10.1038/s41467-022-32312-1)
Supplement: Supplementary file 3 — Reporting Summary [file 41467_2022_32312_MOESM3_ESM.pdf]

nature portfolio

Corresponding author(s): Laurent Renia

Last updated by author(s): 01/06/2022

Reporting Summary

Nature Portfolio wishes to improve the reproducibility of the work that we publish. This form provides structure for consistency and transparency in reporting. For further information on Nature Portfolio policies, see our Editorial Policies and the Editorial Policy Checklist.

Statistics

For all statistical analyses, confirm that the following items are present in the figure legend, table legend, main text, or Methods section.

n/a	Confirmed	
☐	☒	The exact sample size (*n*) for each experimental group/condition, given as a discrete number and unit of measurement
☐	☒	A statement on whether measurements were taken from distinct samples or whether the same sample was measured repeatedly
☐	☒	The statistical test(s) used AND whether they are one- or two-sided<br>*Only common tests should be described solely by name; describe more complex techniques in the Methods section.*
☒	☐	A description of all covariates tested
☐	☒	A description of any assumptions or corrections, such as tests of normality and adjustment for multiple comparisons
☐	☒	A full description of the statistical parameters including central tendency (e.g. means) or other basic estimates (e.g. regression coefficient) AND variation (e.g. standard deviation) or associated estimates of uncertainty (e.g. confidence intervals)
☐	☒	For null hypothesis testing, the test statistic (e.g. *F*, *t*, *r*) with confidence intervals, effect sizes, degrees of freedom and *P* value noted<br>*Give P values as exact values whenever suitable.*
☒	☐	For Bayesian analysis, information on the choice of priors and Markov chain Monte Carlo settings
☒	☐	For hierarchical and complex designs, identification of the appropriate level for tests and full reporting of outcomes
☒	☐	Estimates of effect sizes (e.g. Cohen's *d*, Pearson's *r*), indicating how they were calculated

Our web collection on statistics for biologists contains articles on many of the points above.

Software and code

Policy information about availability of computer code

Data collection	No specific software were used. Data were compiles using excel software (Microsoft).
Data analysis	Data from fluorospot analysis, flow cytometry, cell based inhibition assay and serological assays for detection of SARS-CoV-2 specific antibodies were analyzed with Graphpad Prism software (verision 9). Flow jo software (version 10) was used to analyzed flow cytometry data; Mabtech IRIS Immunospot reader ApexTM Software

For manuscripts utilizing custom algorithms or software that are central to the research but not yet described in published literature, software must be made available to editors and reviewers. We strongly encourage code deposition in a community repository (e.g. GitHub). See the Nature Portfolio guidelines for submitting code & software for further information.

Data

Policy information about availability of data

All manuscripts must include a data availability statement. This statement should provide the following information, where applicable:

- Accession codes, unique identifiers, or web links for publicly available datasets
- A description of any restrictions on data availability
- For clinical datasets or third party data, please ensure that the statement adheres to our policy

The source data for all figures are provided as a Source Data file. Raw data for the flow cytometry or ELISPOT can be obtained upon request to the corresponding author.

Field-specific reporting

Please select the one below that is the best fit for your research. If you are not sure, read the appropriate sections before making your selection.

☒ Life sciences ☐ Behavioural & social sciences ☐ Ecological, evolutionary & environmental sciences

For a reference copy of the document with all sections, see nature.com/documents/nr-reporting-summary-flat.pdf

Life sciences study design

All studies must disclose on these points even when the disclosure is negative.

Sample size	Sampling took place during a defined period (January 2021 - May 2021). These dates were selected based on the timing of ethical approvals and the timescale needed to collect samples. As this was an observational study, no sample size analysis was performed.
Data exclusions	No data were exclude from thee paper. In some experiments (T and B cell analysis) we used a smaller number of individuals due to blood sample availability or cell quality or quantity post processing.
Replication	All experiments depicted included at least two to three biological replicates and all biological replicates were successful.
Randomization	No randomization was done. In some of our analysis we used linear regression model. Randomization was not applicable for this study since we include "all" volunteers prospectively
Blinding	Not applicable - observational study

Reporting for specific materials, systems and methods

We require information from authors about some types of materials, experimental systems and methods used in many studies. Here, indicate whether each material, system or method listed is relevant to your study. If you are not sure if a list item applies to your research, read the appropriate section before selecting a response.

Materials & experimental systems

n/a	Involved in the study	
☐	☒	Antibodies
☐	☒	Eukaryotic cell lines
☒	☐	Palaeontology and archaeology
☒	☐	Animals and other organisms
☐	☒	Human research participants
☒	☐	Clinical data
☒	☐	Dual use research of concern

Methods

n/a	Involved in the study	
☒	☐	ChIP-seq
☐	☒	Flow cytometry
☒	☐	MRI-based neuroimaging

Antibodies

Antibodies used

All antibodies used :
-in flow cytometry in the SFB assay are: .
Anti-human IgG Alexa Fluor 647 (Thermo Fisher Scientific Cat# A21445; RRID:AB_2535862); anti-human IgM Alexa Fluor 647 (Thermo Fisher Scientific Cat# A21249; RRID:AB_2535817); anti-human IgA Alexa Fluor 647 (BioLegend Cat# 411502; RRID:AB_2650697); anti-mouse IgG Alexa Fluor 647, (Thermo Fisher Scientific Cat# A21235; RRID:AB_2535804); anti-human IgG1 (Thermo Fisher Scientific Cat# MA1-34581; RRID:AB_11004658); anti-human IgG2 (BioLegend Cat# 411102; RRID:AB_2686940); anti-human IgG3 (BioLegend Cat# 411302; RRID:AB_2686942); anti-human IgG4 (Thermo Fisher Scientific Cat# A10651; RRID:AB_2534053); anti-spike monoclonal antibody (Thermo Fisher Scientific Cat# 703958; RRID:AB_2866477).

-in B cell Elispot
Antibodies were used as in recommended as in the Human IgG ALP kit was used (#3629-1, Mabteck)

-in T cell Elispot:
Human CD8 and CD4 T cell responses were measured using Human IFN-γ/IL-2 FluoroSpot PLUS kits as per manufacturer's protocol (Mabtech, Sweden). Pre-coated antibodies: IFN-γ mAb (1-D1K), IL-2 mAb (MT2A91/2C95), IL-4 mAb (IL4-I), IL-5 mAb (TRFK5) and IL-13 mAb (MT1318) . Detecting antibodies: for IFN-γ (anti-BAM-490), IL-2 (SA-550), anti-IL4 mAb (IL4-II), anti-IL5 mAb (5A10) and anti-IL13 mAb (25K2). Stimulating antibodies against human CD3 (mAb CD3-2 at 0.02µg/mL) or human CD28 (mAb CD28A at 0.1µg/mL) were also used.

Validation

All antibodies used in flow cytometry study are commercially available, and all have been validated by the manufacturers [Mabtech (https://www.mabtech.com/frequently-asked-questions/antibodies-faqs), ThermoFischer (https://www.thermofisher.com/sg/en/

home/life-science/antibodies/invitrogen-antibody-validation.html), BioLegend (https://www.biolegend.com/en-us/quality/product-development)]and used by other publications (See for Flow cytometry: Goh et al, Cel Rep Med, 2021; for T cell Elispot: Jergovic et al, J Immunol, 2022]. Likewise, we titrated these antibodies according to our own our staining conditions.

Eukaryotic cell lines

Policy information about cell lines

Cell line source(s)	HEK-293T (ATCC CRL-3216), VERO E6 (ATCC CRL-1586), CHO cells (ATCC CCL-185)
Authentication	All cells were originally obtained from a verified vendor that sell authenticated cell line (ATCC). The replicates of each experiments were conducted with cells from different passages and no significant difference was observed between the replicates. No additional authetication was performed.
Mycoplasma contamination	All the cell used in this study are tested regularly for Mycoplasma contamination by PCR and were all negative
Commonly misidentified lines (See ICLAC register)	The cell lines are not listed as commonly misidentified in the ICLAC register

Human research participants

Policy information about studies involving human research participants

Population characteristics	Population characteristics are provided in Supplementary Table 1.
Recruitment	Patients were recruited in the National Center for Infectious Diseases and participating Hospitals. Recruitment for this study was; 18 years of age and older. Able to read, speak and understand English and in good general health.
Ethics oversight	The study design and protocol for the COVID-19 PROTECT study group were assessed by National Healthcare Group (NHG) Domain Specific Review Board (DSRB) and approved under study number 2012/00917. Collection of healthy donor samples was approved by SingHealth Centralized Institutional Review Board (CIRB) under study number 2017/2806 and NUS IRB 04-140. Written informed consent was obtained from all study participants in accordance with the Declaration of Helsinki for Human Research. The experiments adhered to the principles set out in the Department of Health and Human Services Belmont Report.

Note that full information on the approval of the study protocol must also be provided in the manuscript.

Flow Cytometry

Plots

Confirm that:

☒ The axis labels state the marker and fluorochrome used (e.g. CD4-FITC).

☒ The axis scales are clearly visible. Include numbers along axes only for bottom left plot of group (a 'group' is an analysis of identical markers).

☒ All plots are contour plots with outliers or pseudocolor plots.

☒ A numerical value for number of cells or percentage (with statistics) is provided.

Methodology

Sample preparation	Blood was collected in VACUETTE EDTA tubes (Greiner Bio, #455036) or in Cell Preparation Tubes (CPT) (BD, #362761) for volunteers at various timepoints. Cells were seeded at 1.5 x 105 cells per well in 96 well V-bottom plates. Cells were incubated with human serum (diluted 1:100 in 10% FBS) followed by a secondary incubation with a double stain, comprising Alexa Fluor 647-conjugated anti-human IgM, IgG or IgG isotypes (1:600 dilution) and propidium iodide (PI; 1:2500 dilution). All Ig were obtained from Thermo Fisher Scientific: antii Human IgM coupled to Alexa Fluor 647 (#Cat# A21445; RRID:AB_2535862), anti-human IgM Alexa Fluor 647 (Cat# A21249; RRID:AB_2535817), Anti-human IgG1 (Cat# MA1-34581; RRID:AB_11004658), anti-human IgG2 (Cat# 411102; RRID:AB_2686940), anti-human IgG3 (Cat# 411302; RRID:AB_2686942) mouse antibodies. Alexa Fluor 647-conjugated goat anti-mouse IgG were used as secondary antibodies (Cat# A28181).
Instrument	BD Biosciences LSR4 laser
Software	Flow Jo version 10
Cell population abundance	>10e5 per sample
Gating strategy	For the SFB assay, all details have been extensively described in Goh et al (Star Protocols, 2021, 2, 100671). The cells are gated based on the following: Forward (FSC) and side (SSC) scatter parameters, FSC-A/SSC-A, to exclude cell debris. Then, FSC-A/FSC-H rare used to select for single cells . Next, FSC-A/PI are used to select for live cells (PI-negative population; PI is used to exclude dead cells). Gate 2 determined cells positiive FITC/Alexa Fluor 647. Binding is determined by the percentage

of GFP-positive S protein-expressing cells that are bound by specific antibody, indicated by the events that are Alexa Fluor 647- and FITC-positive. We have added the supplementary Figure 5 describing the gating strategy.

☒ Tick this box to confirm that a figure exemplifying the gating strategy is provided in the Supplementary Information.
